# Spontaneous Shrinking and Growing Skull Base Chordoma

**DOI:** 10.1055/a-2587-6573

**Published:** 2025-05-09

**Authors:** Esmée M. Bosman, Max E. Keizer, Jasper van Aalst, Martinus P.G. Broen, Alida A. Postma, Astrid I.P. Vernemmen, Henricus P.M. Kunst, Yasin Temel

**Affiliations:** 1 Department of Neurosurgery, Maastricht University Medical Center+, Maastricht, The Netherlands; 2 Dutch Academic Alliance Skull Base Pathology, Maastricht University Medical Center+ and Radboud University Medical Center, Maastricht and Nijmegen, The Netherlands; 3 School for Mental Health and Neuroscience, Faculty of Health, Medicine and Life Sciences, Maastricht University, The Netherlands; 4 Department of Neurology, Maastricht University Medical Center+, Maastricht, The Netherlands; 5 Department of Radiology, Maastricht University Medical Center+, Maastricht, The Netherlands; 6 Department of Pathology, Maastricht University Medical Center, Maastricht, The Netherlands; 7 Department of Otorhinolaryngology, Radboud University Medical Center, Nijmegen, The Netherlands; 8 Department of Otorhinolaryngology, Maastricht University Medical Center+, Maastricht, The Netherlands; 9 Istanbul Atlas University, Faculty of Medicine, Istanbul, Türkiye

**Keywords:** skull base chordoma, chondrosarcoma, spontaneous shrinkage, hemorrhage, endoscopic, skull base surgery

## Abstract

**Background:**

Chordomas are rare slow-growing tumors occurring in the axial skeleton and can demonstrate local aggressive behavior, typically extending from the median axis, compressing surrounding tissue. Complete surgical resection and adjuvant radiotherapy are the preferred treatments. We present an unusual case of a spontaneously shrinking and growing off-midline petroclival chordoma.

**Case Description:**

A 23-year-old woman presented with right abducens nerve palsy. Computed tomography and magnetic resonance imaging (MRI) revealed an off-midline petroclival lesion compressing the abducens nerve with characteristics of a chondrosarcoma. Preoperative MRI indicated spontaneous lesion regression, and the abducens nerve showed clinical improvement. Hence, the planned surgery was canceled. During the wait-and-scan period, abducens nerve palsy recurred. MRI confirmed lesion growth and showed an intratumoral linear structure indicative of blood. Even though preoperative MRI again demonstrated shrinkage, the lesion was surgically removed. Despite the unusual presentation, histopathological examination diagnosed a conventional chordoma. A second surgery was required to remove the residual tumor, after which the patient received high-dose photon beam therapy.

**Conclusion:**

This article discusses the uncommon presentation and behavior of a petroclival chordoma, showing fluctuating cycles of off-midline growth and spontaneous regression. While intratumoral hemorrhage is hypothesized to explain this tumor behavior, the exact etiology needs further investigation. The case presented here emphasizes the importance of considering chordoma in the differential diagnosis despite an atypical disease course.

## Introduction

Chordomas are rare malignant bone tumors originating from primitive notochordal remnants. 1 2 3 This malignancy can arise anywhere along the craniospinal axis: skull base (32%), mobile spine (32.8%), and sacral (29.2%). 4 Skull base chordomas show an annual incidence of one case per 2,000,000 individuals, constituting less than 0.2% of all intracranial neoplasms. 1 5 Clival chordomas typically show slow growth from the clival midline but can demonstrate locally osteolytic, aggressive behavior, and may compress surrounding neurovascular structures. 6 The long-term prognosis of skull base chordomas is unfavorable, with 5- and 10-year recurrent rates of 52.9 and 88.3%, 7 respectively, and 5-, 10-, and 20-year survival rates dropping to 67.6, 39.9, and 13.1%, respectively. 4 While chordomas are often not metastatic on presentation, 5 to 30% show metastatic potential. 8 9 10 11 12 Gross total surgical resection of the tumor followed by high-dose radiotherapy is considered the gold standard treatment. 6 Here, we describe an unusual disease course of an off-midline, petroclival chordoma.

## Case Presentation

**Fig. 1:**
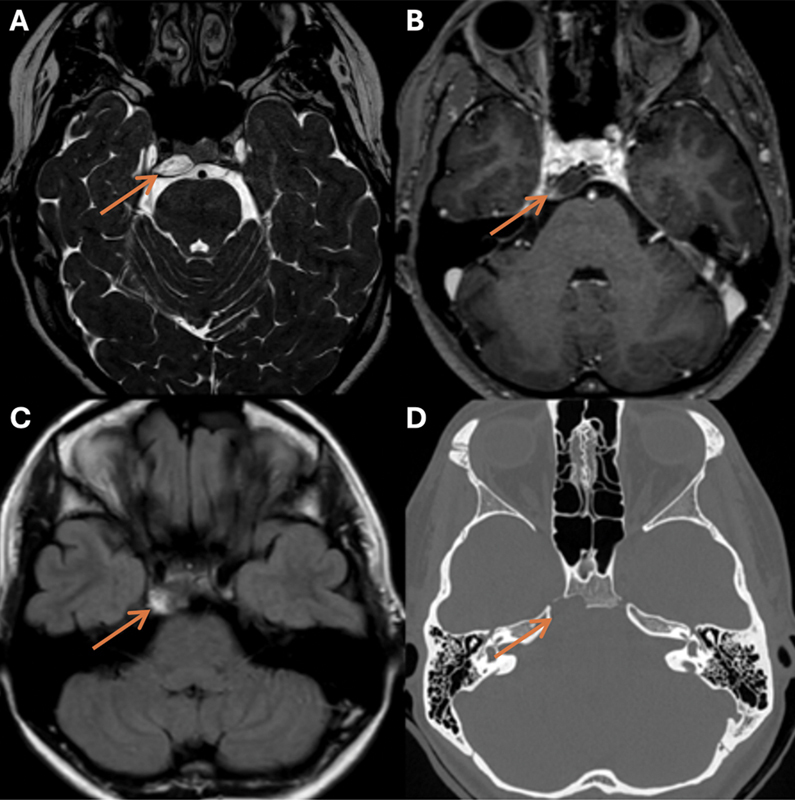
Imaging at presentation demonstrating a lesion at the right petroclival fissure measuring 9 mm × 15 mm × 17 mm (AP; transverse; CC) (orange arrows). The lesion is hyperintense on T2-W ( **A** ), hypointense with only subtle rim enhancement on CE-T1-W ( **B** ), slightly hyperintense on FLAIR ( **C** ), and lytic on CT images with sclerosis of the rim ( **D** ). AP, anterior–posterior; CC, craniocaudal; CE-T1-W, contrast-enhanced T1-weighted, CT, computed tomography; FLAIR, fluid-attenuated inversion recovery; T2-W, T2-weighted.

**Fig. 2:**
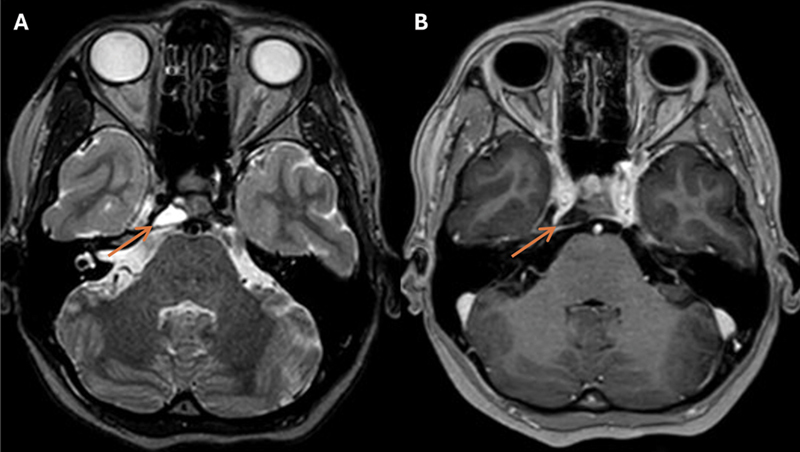
Preoperative neuronavigation imaging, two months after initial presentation, demonstrates decreased AP dimensions, measuring 7 mm × 15 mm × 17 mm (AP; transverse; CC), and an increased distance between the lesion and the pons on T2-W ( **A** ) and CE-T1-W images ( **B** ). AP, anterior–posterior; CC, craniocaudal; CE-T1-W, contrast-enhanced T1-weighted; T2-W, T2-weighted.

A 23-year-old female patient presented with horizontal diplopia due to progressive right abducens nerve palsy. A dull headache with fluctuating intensity and nausea accompanied the developing diplopia but gradually decreased after a few days. The patient did not suffer from other neurological deficits, complaints, or comorbidities. A computed tomography (CT) scan demonstrated an intraosseous lytic lesion at the right petroclival fissure and posterior clivus. Magnetic resonance imaging (MRI) revealed a hyperintense lesion with faint peripheral contrast-enhancing on T2-weighted (T2-W) and a hyperintense signal on fluid-attenuated inversion recovery (FLAIR) images, measuring 9 mm × 15 mm × 17 mm (anterior–posterior, transverse, craniocaudal) with abducens nerve compression at the right canal of Dorello, and intradural expansion in the posterior fossa without brain stem compression ( Fig. 1 ). The differential diagnosis included chondrosarcoma, epidermoid, ecchordosis physaliphora, benign notochordal cell tumor, and off-midline chordoma. Endoscopic transsphenoidal resection was planned 1 month later. The preoperative navigation MRI showed spontaneous regression of the lesion with dissociation from the abducens nerve ( Fig. 2 ), and the patient experienced abduction improvement of the right eye. The differential diagnosis was reconsidered, and a simple bone cyst was added. While simple bone cysts in the skull base are rare, 13 14 15 their spontaneous resolution has been reported in long bones and the mandible. 16 17 Therefore, the procedure was canceled, and a conservative wait-and-scan policy was chosen with a 6-month interval.

**Fig. 3:**
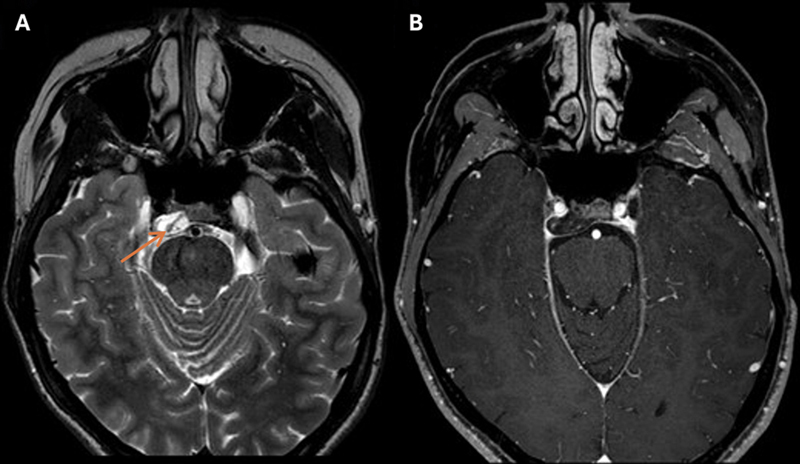
T2-W ( **A** ) and CE-T1-W ( **B** ) imaging 9 months after the canceled surgery demonstrates lesion progression with a hypointense structure traversing the lesion (orange arrow). The lesion measures 9 mm × 19 mm × 17 mm (AP; transverse; CC). anterior–posterior; CC, craniocaudal; CE-T1-W, contrast-enhanced T1-weighted; T2-W, T2-weighted.

At the next follow-up, MRI showed a slight enlargement of the petroclival lesion toward the right abducens nerve without clinical changes, and a 3-month interval wait-and-scan was scheduled. During the waiting period, the right abducens nerve palsy recurred. MRI demonstrated significant lesion growth, affecting the abducens nerve. A hypointense linear structure traversing the lesion was recognized on T2-W images, which could indicate blood ( Fig. 3 ). At this moment, hemangioma, simple bone cyst, and chondrosarcoma constituted the differential diagnosis. Surgery was scheduled.

**Fig. 4:**
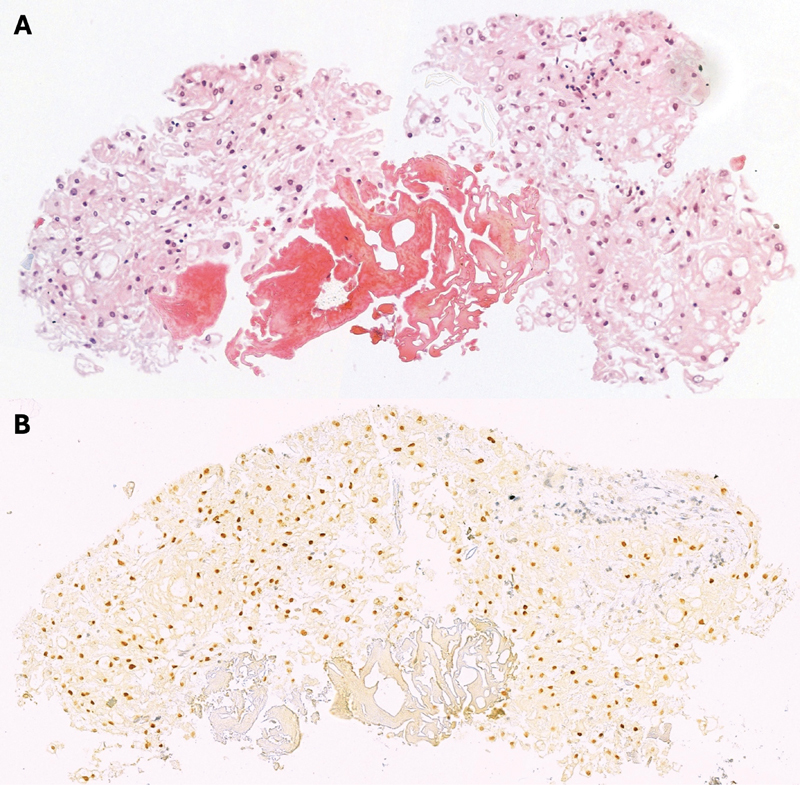
Immunohistochemical staining of the chordoma. Hematoxylin and eosin (H&E) staining (magnification ×20) demonstrates disorganized physaliphorous cells ( **A** ). Tumor cells show nuclear brachyury expression (magnification ×20) ( **B** ).

Interestingly, within a 1-month interval, preoperative MRI again showed lesion regression, consistent with the diplopia improvement experienced by the patient. Nevertheless, the patient underwent an uncomplicated endoscopic endonasal skull base surgery to decompress the abducens nerve further and remove the tumor. Histopathology revealed a population of disorganized physaliphorous cells ( Fig. 4A ). The cell nuclei displayed some anisokaryosis and were normochromic to bright in appearance. Immunohistochemical analysis revealed positive cell staining for cytokeratin AE1/AE3, and a dubiously weak S100 staining for a subset of cells. Additional positive staining for brachyury expression confirmed the diagnosis of a conventional chordoma ( Fig. 4B ). Next to the tumor was a small focus of degenerated erythrocytes, yet pre- versus intraoperative hemorrhage could histologically not be distinguished with certainty.

While postoperative MRI showed an evident size reduction of the petroclival lesion, a minor residual persisted dorsal to the right internal carotid artery. A second endoscopic endonasal surgery was performed for maximal, although safe, resection of the residual lesion. Postoperative MRI indicated no macroscopically evident lesion remnants. Subsequently, the patient received photon beam therapy with a dose of 74 Gy in 37 sessions. Abducens nerve function largely recovered. At 1-year follow-up, the patient is recurrence-free.

## Discussion

Chordomas are rare neoplasms that typically show progressive growth and locally aggressive behavior. Here, we describe an unusual disease course of a skull base chordoma, which showed off-midline growth, spontaneous shrinkage without intervention, and signs of a small hemorrhage. While lesion growth induced abducens nerve palsy, shrinkage led to abducens and diplopia recovery.

Clival chordoma diagnosis depends on clinical presentation, histopathology, and imaging characteristics associated with the tumor location and invasion. 18 CT and MRI are the preferred imaging modalities to assess bone involvement and evaluate the proximity of the lesion to critical soft tissue structures. On non-contrast CT, clival chordomas often appear as osteolytic lesions that are well-circumscribed, hypoattenuating, and heterogeneous. 19 20 The tumor bulk generally has increased density compared with surrounding neural structures. 19 Sometimes, CT shows intratumoral calcifications or areas of low attenuation representing mucinous content within the tumor. 19 20 On MRI, chordomas appear hypointense on T1- and hyperintense on T2-W images. This signal intensity may vary with foci of hyper- or hypointensity on the respective MRI sequences, resembling a heterogeneous honeycomb enhancement pattern related to mucus, hemorrhage, or calcification. 20 21 Gadolinium administration usually leads to mild heterogeneous contrast enhancement with restricted diffusion-weighted imaging. 7 These CT and MRI features are in line with the presented case.

However, chondrosarcomas share similar characteristics in both imaging modalities, complicating their distinction from chordomas on preoperative imaging. 20 22 The location of the tumor origin is often used as a differentiation direction. Skull base chordomas usually grow in the clival midline, while a lateral clival origin, in and next to the petroclival fissure, would favor chondrosarcoma. 20 23 Yet, chordomas growing laterally in the petrous bone have previously been described. 24 As for the presented case, immunohistochemical detection of brachyury is currently required to confirm the diagnosis of chordoma.

Only a few cases of spontaneous chordoma regression are known: two in the skull base, 25 26 one in the cervical region, 27 one in the sacrococcygeal region, 28 and pulmonary metastases of a sacral chordoma. 29 Bander et al 25 described a case of spontaneous clival chordoma regression without surgical intervention or radiotherapy. Herbal supplements and animal oils consumed by the 75-year-old female patient were hypothesized to exert antitumoral effects and contribute to tumor shrinkage. 25 Radl et al 27 reported a case of chordoma disappearance in the C2 vertebral body after nonoperative treatment in a 24-year-old male patient. The patient received dexamethasone and antibiotic treatment, of which the immune-mediated mechanisms were proposed to contribute to chordoma disappearance. 27 However, similar to the case presented here, the remaining cases do not share these inflammatory or immunological factors. 26 28 29 Instead, González et al 28 related the spontaneous regression of a sacrococcygeal chordoma to intratumoral hemorrhage resorption. Intratumoral foci of hemorrhage can be confirmed by hypointensity on susceptibility-weighted imaging. 18

Hemorrhagic chordomas are uncommon but have previously been documented, mainly in clival chordomas. 18 25 While the underlying etiology remains elusive, some mechanisms have been suggested. Rapid tumor growth could lead to an inadequately matched blood supply and rupture of fragile vessels. 18 Similarly, small vessel occlusion after vascular proliferation can result in areas of ischemic necrosis and hemorrhage. 18 30 Moreover, chordomas may induce neovascularization, resulting in increased vessel permeability and fragility, with spontaneous hemorrhage as a consequence. 31 These phenomena seem to correlate with vascular endothelial growth factor (VEGF) overexpression occurring in chordomas. 31 32 VEGF is known for inducing tumor angiogenesis and is often hypoxia-driven. 32 33 Indeed, the presence of hypoxic areas in chordomas has been presented by [18F]-fluoromisonidazole positron emission tomography/CT. 34 35 Hypoxic tumor areas are generally radioresistant and may indicate poor prognosis. 19

For the case presented here, cycles of intratumoral hemorrhage and partial hemorrhage resolution are hypothesized to explain the fluctuating tumor growth and shrinkage. The hyperintensity on the first FLAIR MRI and the linear structure observed on the T2-W MRI may have indicated the occurrence of intratumoral hemorrhage that led to temporally increased tumor size. The linear structure was only observed after tumor growth but disappeared after shrinkage. Yet, the underlying mechanism driving these processes remains unknown. Various factors may be involved in this odd disease course, and further research is required to evaluate the etiology.

## Conclusion

To conclude, we present an unusual case of chordoma growth and spontaneous regression. While no consensus has yet been achieved on the underlying mechanisms of growth reversibility, this odd disease course should increase awareness and warrant further investigation into chordoma behavior. Off-midline presentation, intratumoral hemorrhage, and spontaneous regression should not exclude chordoma from the differential diagnosis or change the treatment. Hence, histological confirmation is mandatory in such cases.
